# Real-Time Analysis of Neuronal Cell Cultures for CNS Drug Discovery

**DOI:** 10.3390/brainsci14080770

**Published:** 2024-07-30

**Authors:** Millicent T. Akere, Kelsee K. Zajac, James D. Bretz, Anvitha R. Madhavaram, Austin C. Horton, Isaac T. Schiefer

**Affiliations:** 1 Department of Medicinal and Biological Chemistry, College of Pharmacy and Pharmaceutical Sciences, University of Toledo, Toledo, OH 43614, USA; millicent.akere@rockets.utoledo.edu (M.T.A.); kelsee.zajac@rockets.utoledo.edu (K.K.Z.); james.bretz@utoledo.edu (J.D.B.); anvitha.madhavaram@rockets.utoledo.edu (A.R.M.); austin.horton@rockets.utoledo.edu (A.C.H.); 2 Center for Drug Design and Development, College of Pharmacy and Pharmaceutical Sciences, University of Toledo, Toledo, OH 43614, USA

**Keywords:** IncuCyte, neuronal cell culture, in vitro drug development, live-cell imaging, methods

## Abstract

The ability to screen for agents that can promote the development and/or maintenance of neuronal networks creates opportunities for the discovery of novel agents for the treatment of central nervous system (CNS) disorders. Over the past 10 years, advances in robotics, artificial intelligence, and machine learning have paved the way for the improved implementation of live-cell imaging systems for drug discovery. These instruments have revolutionized our ability to quickly and accurately acquire large standardized datasets when studying complex cellular phenomena in real-time. This is particularly useful in the field of neuroscience because real-time analysis can allow efficient monitoring of the development, maturation, and conservation of neuronal networks by measuring neurite length. Unfortunately, due to the relative infancy of this type of analysis, standard practices for data acquisition and processing are lacking, and there is no standardized format for reporting the vast quantities of data generated by live-cell imaging systems. This paper reviews the current state of live-cell imaging instruments, with a focus on the most commonly used equipment (IncuCyte systems). We provide an in-depth analysis of the experimental conditions reported in publications utilizing these systems, particularly with regard to studying neurite outgrowth. This analysis sheds light on trends and patterns that will enhance the use of live-cell imaging instruments in CNS drug discovery.

## 1. Introduction

### 1.1. CNS Drug Discovery Challenges

The central nervous system (CNS), which comprises the brain and spinal cord, is undoubtedly the most delicate system in the human body, owing to its susceptibility to damage and injury. Compared to other tissues and cells, the regenerative capacity of neurons in the central nervous system is restricted; hence, their difficulty in repairing and regenerating after damage [1]. Brain diseases or disorders include, but are not limited to, psychiatric disorders, neurodegenerative diseases, brain cancers, and stroke [2]. With respect to other disease areas, developing new therapeutics for CNS disorders poses distinctive difficulties due to the intricate interactions of the brain’s molecular and cellular components [3]. One of these cellular components is the blood–brain barrier (BBB), which impedes the transport of many drugs from the bloodstream to the brain, thereby diminishing their efficacy. Neurodegeneration remains a hallmark of a multitude of untreatable CNS diseases, such as Parkinson’s disease (PD), Huntington’s disease (HD), Alzheimer’s disease (AD), and amyotrophic lateral sclerosis (ALS). The prevalence of these diseases continues to rise as the population begins to age, and because of the elusive nature of their pathogenesis, only a few treatments that mitigate their symptoms are available [4,5]. Despite great efforts invested in the CNS drug discovery process, there has been a steady decline in the amount of CNS drugs in clinical trials over the past two decades [3,6,7]. This attrition rate associated with CNS drug discovery as compared to non-CNS drug discovery can be attributed to the challenges associated with the drug discovery process, as several CNS drugs have been discontinued in clinical trials due to their inefficacy [6,7]. This inefficacy is a result of the elusory nature of the disease’s etiology and the absence of well-validated animal models for the diseases [7]. An alternative approach to animal models is the application of neuronal cell cultures as models for CNS drug discovery.

### 1.2. Neuronal Cell Cultures

Neuronal cell culture plays a vital role in CNS drug discovery, as it provides a controlled and reproducible environment to study the effects of potential drug candidates. It is preferred over animal models because of its high-throughput screening compliance, disease modeling feasibility, and the physiological relevance of human-derived neuronal culture [8,9]. Neuronal cell cultures can be categorized into different types based on their sources and applications, but for the purpose of this paper, only three types will be discussed: primary, immortalized/cancer, and stem cell derived cultures. Primary neuronal cultures are derived directly from neural tissues in the brain and spinal cord. This makes them more desirable as they provide a more native environment for studying cellular functions when compared to other cell culture types [10]. Immortalized cell cultures are derived from neuronal tumors, which makes their culturing process relatively easy due to their unlimited proliferation. Whereas stem cells or human induced pluripotent stem cells (hiPSCs) are acquired from somatic cells and then differentiated into neurons, making them great accessories for modeling neurodegenerative diseases [10]. Neurites, which are small processes in neurons, are notably affected by alterations in neuronal homeostasis, which are present in most neurodegenerative disorders [11]. Diminished synaptic connectivity and deterioration of neuronal networks often precede neuronal death, making neurite protection/regeneration an attractive target to ameliorate the progression of these disorders [12,13]. Hence, there is an urgent need to discover small-molecule therapeutics, capable of initiating neuritogenesis. Psychoplastogens, a new class of fast-acting neurotherapeutics, have been implicated in the enhancement of neural plasticity and also in the generation of long-lasting beneficial effects on neurons [14,15]. Thus, screening psychoplastogenic molecules in stem cell-derived disease models will be a step in this journey of CNS drug discovery. Methods to quantify neurite outgrowth or neurite death rely on the examination of fixed cells under a microscope or the measurement of the total amount of fluorescence from labeling [16,17]. Both approaches may result in a single time point since the cells are usually not viable after exposure to stains and dyes. Automated microscopes and related software are capable of quantifying neurite outgrowth; however, such analysis has limited throughput [18,19]. The technique capable of addressing the drawbacks of these conventional methods is the live-cell imaging technique.

### 1.3. Live-Cell Imaging Systems

**Figure 1:**
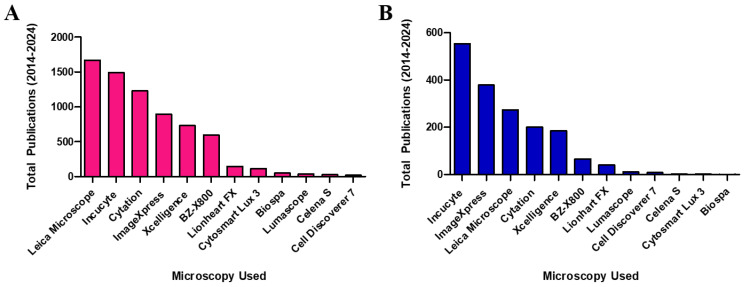
Overview of the types of microscopic imaging devices used in neuroscience. A Google Scholar search was conducted to determine (**A**) The number of publications that utilized each instrument in the field of neuroscience and (**B**) The number of publications that utilized each instrument in neurite outgrowth analysis since 2014.

Live-cell imaging has been around for the last century and has steadily evolved into advanced automated instruments that allow for rapid and accurate data collection [20]. The automation and innovation of these systems offer researchers an efficient approach to studying biological systems in real-time. While these instruments have limitations, they have integrated their way into the study of dynamic cellular events. Traditional methods of cellular imaging typically require fixation and specific staining onto a slide, which ultimately provides a snapshot of an entire experiment. Fixating cells can introduce artifacts and obscure cellular behaviors, potentially confounding experimental results. For instance, Schnell et al. demonstrated that live-cell imaging provides more accurate and reproducible measurements of cellular dynamics compared to fixed-cell assays [21]. Similarly, Cheng et al. emphasized the importance of live-cell analysis in preserving the physiological relevance of cellular responses [22]. By avoiding the fixation process, live-cell imaging systems like the IncuCyte enable real-time observation of neurite outgrowth and other cellular phenomena, providing more reliable data for CNS drug discovery. Although endpoint analyses may provide sufficient evidence for certain readouts, this deviates from the intricacies that are discoverable while cellular processes are taking place. Real-time cellular analysis allows for a greater understanding of biological systems without impacting the system itself. A vast array of live-cell imaging systems currently monitor complex cellular phenomena today, and there is a particular niche for these instruments in the field of neuroscience. Figure 1A illustrates the increasing use of live-cell imaging systems, with IncuCyte systems (Essen Bioscience, Inc., Ann Arbor, MI, USA) being prominently utilized in neurite kinetic assays (Figure 1B). Based on this finding, we decided to focus our review on IncuCyte systems as a representative of live-cell imaging in neuroscience.

This paper reviews the current state of IncuCyte systems and their applications in neuronal cultures. Since the application of these instruments is relatively new, we will highlight some of the technical specifications and summarize the trends in the experimental conditions from relevant publications, particularly those focusing on neurite kinetic assays. While this list is not exhaustive, we believe the trends and data from these papers are representative of the field and can be applied to other live-cell imaging systems. Our goal is to identify trends and recommendations based on published studies to advance live-cell imaging in CNS drug discovery. No formal statistical analysis was performed as our focus was primarily on descriptive and trend analyses.

## 2. Key Features and Components of Live-Cell Imaging

Live-cell imaging has proven to be a game-changer in our quest to unravel the mysteries of cellular life [20]. Its impressive capabilities and wide range of components have opened new avenues of exploration and deepened our understanding of the intricate workings of cells. With its ability to uncover the intricacies of cell division and aid in drug discovery, this technique has transformed biological research and has the potential to unravel the enigma of life itself. The basic features and components of live-cell imaging include but are not limited to, time-lapse imaging and automation, fluorescent labeling, microscopic techniques, and environmental control (Table 1).

Time-lapse imaging is an effective tool for capturing the progression of slow-moving processes over time through a series of images. The subsequent replay of these images occurs at an accelerated rate, revealing unobservable slow processes. Time-lapse imaging is more often combined with automation, which allows for extended experiments without requiring human intervention. The application of fluorescent labeling facilitates the precise and targeted observation of specific cellular components or molecular processes. Upon excitation, these fluorophores, attached to proteins or other targets of interest, emit light, thereby revealing their spatial distribution and locomotion within the cell. Furthermore, the microscope is a fundamental component of any live-cell imaging system. There are different types of microscopy used in live-cell imaging, but the common ones are phase-contrast, differential interference contrast, fluorescent, confocal, and bright field microscopy. Choosing a microscopic method depends on the type of resolution needed, the size of the specimen, and the effect of the modality on the health of the cells. Lastly, environmental control ensures cells remain viable throughout the imaging period by maintaining stable conditions like temperature, humidity, and CO_2_ levels [34,35,36,37,38]. The general features of the live-cell imaging system in Figure 1 are listed in Table 1.

An important consideration for these studies is accessibility, which is related to costs. Simplistically speaking, these systems are cell incubators modified with fairly rudimentary robotics (in some cases) and advanced multimodal imaging microscopes. Each system comes with the unique features and associated capabilities described in Table 1, and the costs of the systems are largely driven by microscopy. For instance, the IncuCyte, offering 5-color fluorescence channels and phase-contrast microscopy, may fall into the moderate to high-cost range, particularly when additional software modules like NeuroTrack are considered. The Leica Microsystems Mica Microhub (Leica Microsystems, Wetzlar, Germany), with 4-color widefield fluorescence and confocal microscopy, is costly due to its advanced imaging capabilities and environmental control features. Agilent BioTek’s Cytation 10 and Lionheart FX (Agilent Technologies, Santa Clara, CA, USA), featuring widefield and spinning disk confocal fluorescence microscopy, along with brightfield and phase-contrast capabilities, are also high-cost due to their comprehensive imaging and live-cell incubation features. In contrast, Agilent’s xCELLigence RTCA eSight (Agilent Technologies, Santa Clara, CA, USA), combining brightfield microscopy with impedance-based cell analysis, is more moderately priced, although higher than basic imaging systems. Molecular Devices’ ImageXpress Pico (Molecular Devices LLC., San Jose, CA, USA), with phase contrast, brightfield, and confocal imaging, is another high-cost option driven by its advanced imaging modalities. Etaluma’s Lumascope (LS720) (Etaluma, Inc., Carlsbad, CA, USA) is more cost-effective, with lower to moderate costs due to external incubation requirements, making it suitable for labs with basic fluorescence and brightfield imaging needs. Zeiss’ Cell Discoverer 7 (ZEISS Group, Oberkochen, Germany), incorporating brightfield, confocal, and widefield fluorescence microscopy with comprehensive environmental control, is very high-cost and ideal for detailed cellular analysis. Lastly, the Keyence BZ-X800 (Keyence Corporation, Osaka, Japan) equipped with fluorescent, brightfield, and phase-contrast microscopy, along with a built-in dark room and time-lapse incubation, also falls into the high-cost category, supporting detailed and long-term live-cell imaging studies. Overall, the costs of these systems are influenced by their advanced imaging capabilities, integration of environmental controls, and additional software requirements. In most instances, the capabilities of the instruments can be tailored to the purchasing agent, which can significantly affect the price of acquisition. For precise budgeting, obtaining tailored quotes from vendors and considering the total cost of ownership, including maintenance and service contracts, are important considerations. Simpler systems generally run between $80–150 k, whereas those with more advanced microscopes can cost upwards of $500 k. For this reason, these types of instruments are usually owned/maintained within imaging/instrumentation cores and are rarely used with sufficient frequency to be justified for purchase by a specific sole investigator.

## 3. In-Depth Analysis of IncuCyte Systems: A Common Live-Cell Imaging System

**Figure 2:**
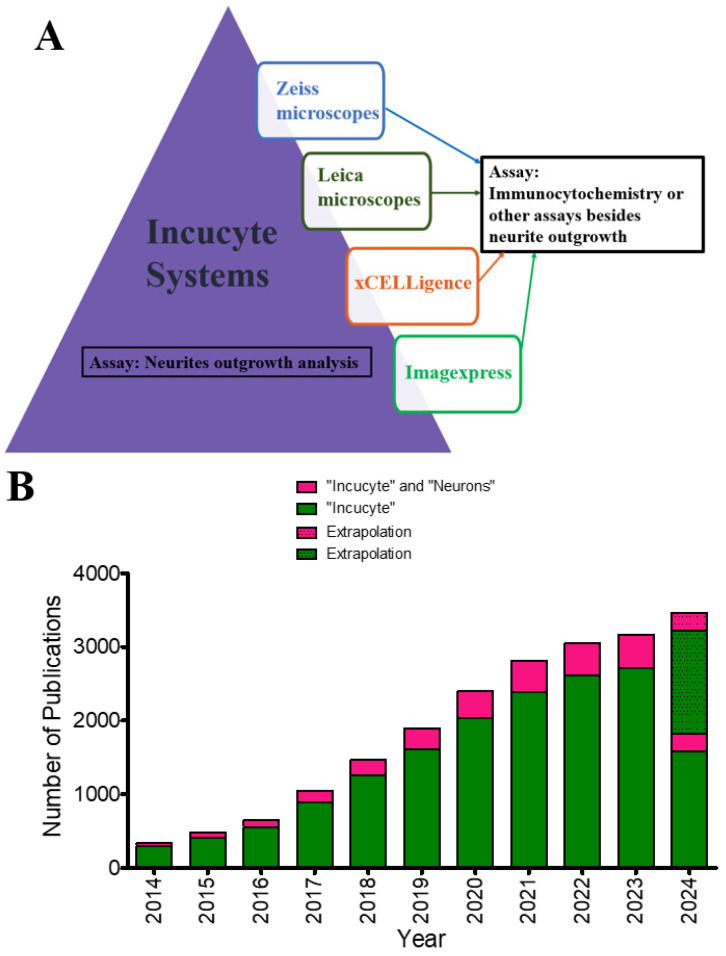
A Google Scholar search of the publications containing terms (**A**) “Incucyte” and “Instrument” and “Neurite Outgrowth.” Note: Instrument refers to either Zeiss microscopes [39,40,41,42,43], Leica microscopes [39,44,45,46], xCELLigence [47,48,49], or ImageXpress [50,51,52]. (**B**) “Incucyte” and “Incucyte” and “Neuron” since 2014. Extrapolation for 2024 was performed by adding the average increase over the years to the total number of publications for 2023 and then subtracting the number of publications so far in 2024 from that number.

After conducting a brief survey of the publications using the top four instruments in Figure 1B, it became evident that many publications referencing the use of any of these four instruments and an IncuCyte instrument utilized these devices primarily for immunocytochemistry or immunohistochemistry imaging, whereas IncuCyte was used in live-cell imaging of neurite outgrowth (Figure 2A). In addition, the prevalence of IncuCyte systems in neuroscience has shown significant growth over the past decade, as evidenced by the consistent increase in the number of publications mentioning their use in neurite outgrowth assays (Figure 2B). This analysis strongly supports the conclusion that IncuCyte systems are predominantly utilized in neurite kinetic assays, highlighting the necessity for a comprehensive examination of IncuCyte systems.

### 3.1. Specifications and Features

Sartorius, the manufacturer of IncuCyte Systems, has several live cell analysis systems. These systems are composed of a phase-contrast microscope with two to five color fluorescent channels and a digital camera. The microscope is placed inside an incubator that maintains cell culturing conditions while allowing frequent imaging and remote observation of cultures throughout an experiment. A critical advantage of live-cell imaging systems is the ability to continually monitor live cells without disruption of the environment. Such disruptions can alter experimental results and detract from the physiologically relevant environment in which the cells are grown. While capturing the images, the plates remain stationary, and the optics move around, minimizing any disturbance usually seen in other approaches to image acquisition. These instruments include several microscope objectives (4×, 10×, and 20×) that increase the range of images acquired and have the capability to analyze at least six-well plates. These six spots are compatible with several types of well plates, allowing for more variety and a higher throughput.

Software applications such as area determination (confluency) and object counting are found in their “basic” analysis package. Additional software components can be added for specific applications that range from chemotaxis migration quantification to the quantification of neurite outgrowth, to name a few. One of the additional software modules available is called NeuroTrack. With this addition, neurite length can be measured by using phase-contrast images that do not disturb the culture when captured. The software includes a mask algorithm that overlays and quantifies neurite dynamics. It can be fine-tuned by the user to ensure the correct coverage and measurement of neurites.

### 3.2. Analysis of Publications Utilizing IncuCyte in Their Neurite Quantification Methods

**Figure 3:**
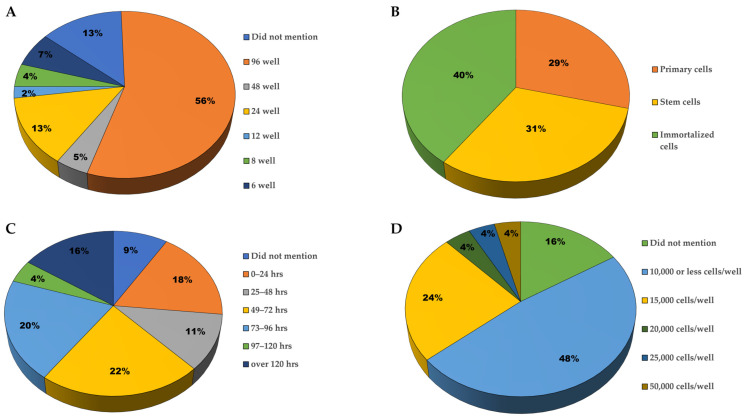
Trends seen in the data of publications that utilized IncuCyte systems in their neurite outgrowth analysis. (**A**) Types of well plates used. (**B**) Types of neurons used. (**C**) Imaging duration. (**D**) Density plated in a 96-well plate.

We analyzed the experimental conditions of 45 papers (Table 2), some of which were highlighted by Sartorius on their publication page. Figure 3 depicts a summary of the analyses performed on each of these articles. These publications range from the year 2014 to present.

#### 3.2.1. Plating Characteristics

Depending on the readout of the assay, the choice of well plate and the density of neurons plated can play a major role in the experimental setup [93]. IncuCyte has the compatibility to image a wide array of multi-well plates depending on the throughput and density desired by the researcher. In these publications, a variety of multi-well plates were utilized, and the most used for the neurite outgrowth assay was the 96-well plate (56%; Figure 3A). Based on this high percentage, we can speculate that these labs were trying to avoid the edge effect while maintaining high throughputs in their assays. The edge effect is one of the architects of the discrepancies observed between cells growing in the inner and outer wells of multi-well plates [94]. This can be avoided in a 96-well plate assay by filling the outer wells with phosphate buffered saline (PBS) or extra media, while the inner 60-wells can still be used for the analysis [93,94].

Neurons typically do not grow well in low-density cultures, but this is necessary for assays that focus on neurite morphology [93]. Developing neuronal networks rely heavily on their surrounding environment, which means that cell plating density can affect the morphology of neurites and synaptic density [95,96,97]. A 96-well plate offers an optimal well size for low-density plating and an adequate number of wells per treatment condition. Since neurite length quantification depends on the visualization of individual neurites, it was intuitive that these publications would gravitate toward low-density cultures. This is evidenced by the number of papers that reported their plating of 10,000 or fewer neurons per well in 96-well plates (Figure 3D).

#### 3.2.2. Type of Neurons Used

With a broad range of available neuron cultures, the choice of neuron plated is completely dependent on the model being studied. Based on 45 studies, a myriad of cell types were utilized, but the specific neurons could be placed into three over-arching categories: stem cells, immortalized cells, and primary cells [98]. The primary and stem cell categories were roughly split equally between them, but the immortalized cells had the highest number of publications reported in Figure 3B.

Fourteen of the publications reported using stem cells to study the length of neuronal processes [42,44,46,55,56,57,58,60,63,67,68,70,76,87]. Of these publications, 10 used human induced pluripotent stem cells (hiPSCs) [42,55,56,58,60,63,67,68,70,76]. As mentioned earlier, it is an invaluable tool used to study intricate pathologies commonly seen in neurodegenerative disorders [99]. Although several papers used regular induced neurons, some papers used specialized neurons from hiPSCs to model a specific disease state. For example, in Korecka et al., 2019, researchers used hiPSCs transfected with the LRRK2 G2019S mutation to study the effects of ER calcium control in Parkinson’s disease [58]. In addition, five out of six AD publications also used hiPSCs in their disease models [55,56,60,63,76].

Of the eighteen immortalized cell lines used in these publications, seventeen were cancerous, and the other was an immortalized mouse hippocampal cell line [43,48,64,65,72,73,75,78,80,81,82,83,84,86,89,90,91,92]. Unlike the other two categories, immortalized cell lines, especially cancer cells, are robust and capable of growing almost indefinitely, making them a common cell type used in neurochemical research [98,100,101]. Although ideal for cancer research, cancer cell lines exhibit behaviors different from non-transformed cells, making them a limited resource for studying certain disease states in the brain. Out of the seventeen papers that reported the use of cancer cell lines, five used them to study cancer-specific pathology [65,81,82,83,84], and another six employed them in the studies of the psychoplastogenic nature of six different natural agents [72,73,75,78,91,92]. The six remaining publications used cancer cell lines to study neurotoxicity and neuroinflammation in different neurological diseases [43,48,64,86,89,90], whereas the immortalized cell line was used for cellular differentiation [80].

Primary neuronal cells are obtained directly from an animal or human brain and plated for culture [98]. These cells do not divide and are typically used in short-term experiments [98]. A benefit of primary cell lines is the ability to study the way certain neurons behave in different parts of the brain. This gives researchers an opportunity to focus on disease pathologies that are localized to a specific region [102]. Out of the thirteen publications reporting the use of primary cell cultures, five used primary cortical neurons, and two used rat spinal cord neurons from various species to conduct their experiments [53,54,59,61,74,77,85]. The remaining articles all used location-specific neurons from rodents for their studies [62,66,69,71,79,88]. 

#### 3.2.3. Secondary Readouts and Confirmatory Experiments

In addition to the neurite length quantification studies, several labs reported conducting correlative/secondary experiments to further validate the results they received. There are a variety of techniques to support neurite outgrowth findings, and the most used ones are immunocytochemistry, cell viability assays, and western blots. Cell viability assays have a wide choice of methods, including the MTT assay, Cell Titer-Glo assay, and Ethidium homodimer assay [67,72,79,81,82,83,84]. Labs reporting the use of immunocytochemistry often use staining to evaluate cell health or confirm the action of a particular molecule through antibody-based staining. The most used secondary readout experiment was immunocytochemistry, with 47% of the papers reporting its use (Table 2). In one paper, the researchers used immunofluorescence quantification of neurotrophin-3 (NT-3) to support its role in neurite outgrowth [77]. Another group used a western blot to confirm the over-expression of a protein, KLF7, following transfection with AAV-KLF4; the cells transfected demonstrated an increase in neurite length [61]. There are several ways to correlate the findings from neurite length quantification results, and the most appropriate correlative experiment depends upon the scope and matter of the work.

#### 3.2.4. Types of Microscopy Employed

The two types of microscopy used in IncuCyte systems are phase-contrast and fluorescent microscopy. The phase-contrast method was introduced by Frits Zernike in 1934 [103]. This method of illumination was born out of his desire to improve the visibility of unstained cell components that were transparent under a light microscope [103]. Because the cell does not require staining for the components of the cell to be seen, this method is termed non-invasive. Fluorescent microscopy, on the other hand, is an invasive technique that sometimes requires the use of fluorophores to visualize the presence of certain macromolecules in a cell [104]. Not only is the addition of these fluorophores intrusive, but the excitation light that brings about their fluorescence also causes phototoxicity in live cells [104]. All 45 publications utilized IncuCyte’s phase-contrast microscopy to capture the images of neurites. Some of these articles were not explicit about their use of phase-contrast microscopy, but it could be implied because they used IncuCyte imagers.

**Figure 4:**
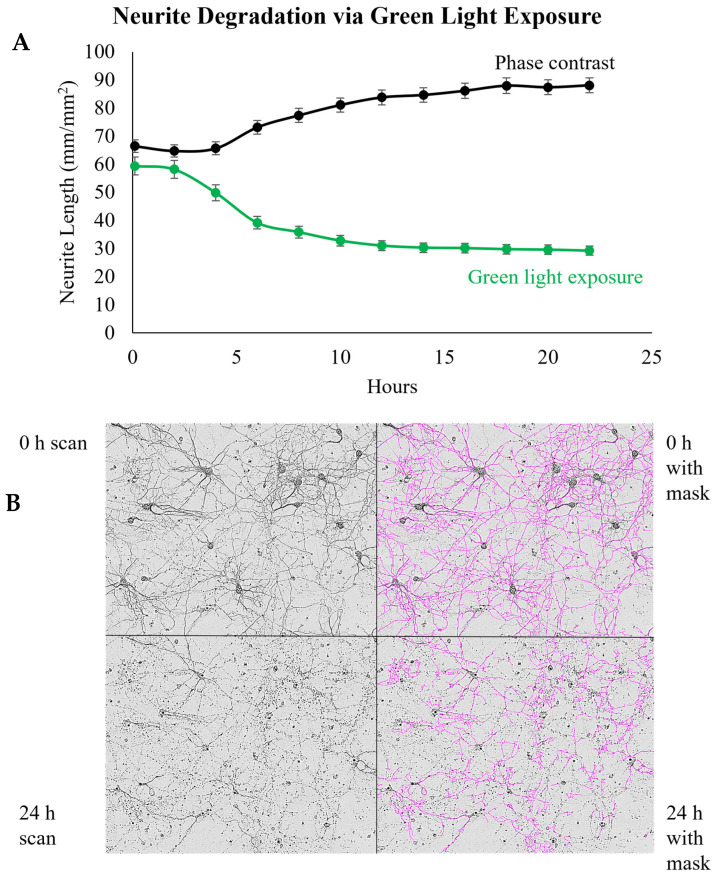
Green light exposure causes the degradation of neurite networks. (**A**) Neurons were cultured for 8 DIV. Media were aspirated and quickly replaced with 50 µL media containing 0.25% DMSO for 24 h to simulate treatments with test compounds. On day 9 DIV, the cultures were placed in the IncuCyte S3 and scanned every 2 h using the Phase and Green (excitation wavelength 440–480 nm) image channels. No green fluorophores were added. Neurite length was determined using the IncuCyte NeuroTrack analysis program. Data are displayed as mean ± SEM (*n* = 48-phase contrast; *n* = 24-green light). (**B**) Micrographs of primary neurons exposed to the green image channel with and without mask coverage.

**Figure 5:**
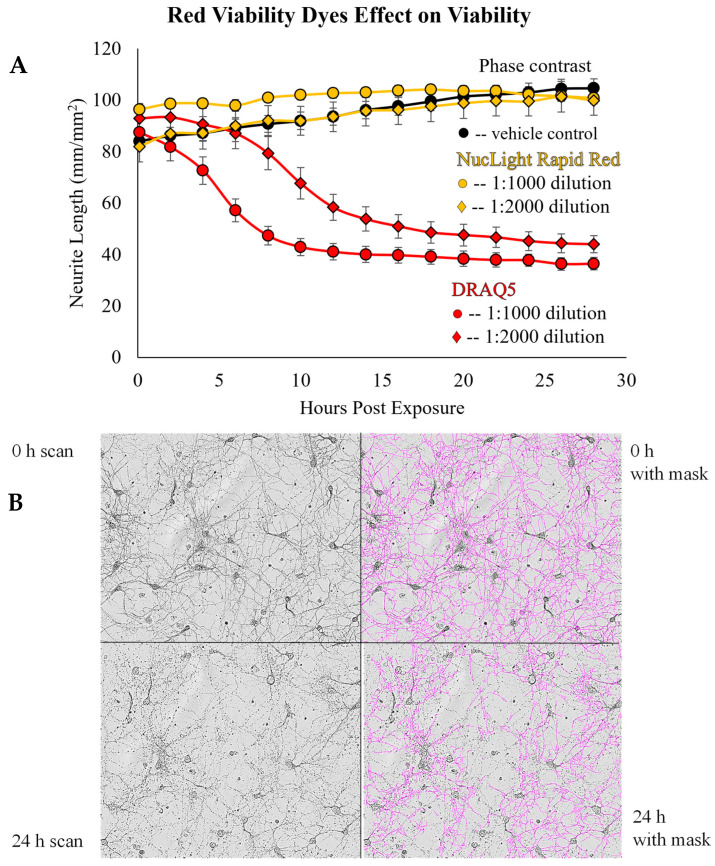
DRAQ5 is cytocidal following red light exposure, while NucLight Rapid Red has no effect on viability. (**A**) On Day 9 DIV, the red fluorophore was added to the cultures to make the final dilution described in the figure legend by adding an additional 50 µL of media containing double the concentration. The cultures were placed in IncuCyte S3 and scanned every 2 h using the Phase and Red (excitation wavelength 565–605 nm) image channels. Neurite length was determined using the IncuCyte NeuroTrack analysis program. Data are displayed as mean ± SEM (*n* = 24-untreated; *n* = 6-treatment groups). (**B**) Micrographs of primary neurons exposed to the red image channel after DRAQ5 exposure, with and without mask coverage.

Furthermore, 18% of the publications employed fluorescent microscopy in their neuronal studies. For instance, Snyder et al. utilized fluorescent microscopy to measure the cell viability of iPSC-derived neurons by adding a green fluorescent dye, CellEvent Caspase 3/7 Green Detection Reagent, to the cells at the start of the experiment and measuring its fluorescence at the end [70]. This fluorescent dye was also tested alone to ensure the reagent was not contributing to the apoptosis measured [70]. In addition, while using the fluorescent microscopy component of this system, we encountered several issues running our neurite outgrowth studies with primary rat cortical neurons. One of the drawbacks is that the green fluorescence excitation wavelength (440–480 nm) kills primary rat cortical neurons (Figure 4). Without the use of this wavelength, we cannot conduct applications like the cell viability assay. Another problem that we faced involved the viability indicator DRAQ5. This is a viability stain using membrane permeability, DNA binding, and red fluorescence to detect live cells [105]. DRAQ5 becomes cytocidal when combined with the excitation wavelength for red fluorescence, but we overcame this issue when we substituted DRAQ5 for the IncuCyte NucLight Rapid Red Reagent (Figure 5).

### 3.3. Imaging Duration

The imaging duration is determined by a lot of factors, including the cell type and the type of analysis. The frequency of imaging is another detail that also needs to be considered. The higher the frequency, the better the understanding of the biological process being studied; this is due to the slow tendencies of cell processes [106]. On the other hand, the higher the frequency of imaging, the more susceptible the cells are to phototoxicity if they are under illumination [106]. Nevertheless, the highest frequency of imaging that cells endure under experimental conditions can be determined by adjusting the time period between successive images [106]. This optimization process was observed in three of these publications. Here, the authors started out by imaging every 6 h (h) for 5 days, then every 6 h for 4 days, and then every 12 h for 4 days [81,82,83,84]. IncuCyte systems can be used to monitor neurite outgrowth for hours, days, weeks, or even up to a month. Figure 3C illustrates the trend in imaging duration observed across these publications; the longest duration was 450 h [57].

## 4. Discussion and Future Directions

The challenges in CNS drug discovery are multifaceted owing to the complexity of brain physiology and the limited regenerative capacity of neurons. Traditional drug discovery approaches have faced significant hurdles, leading researchers to explore alternative approaches. Neuronal cell cultures coupled with live-cell imaging systems have emerged as indispensable tools for this endeavor, offering controlled and reproducible environments for studying the effects of potential drug candidates. In a Google Scholar search of twelve live-cell imaging devices and the publications that utilize them in neuroscience, it was discovered that Leica microscopes were the most frequently used imaging devices among the twelve. However, when this search was narrowed down to publications that studied neurite outgrowth, IncuCyte was determined to be the most often used device (Figure 1).

IncuCyte systems have gained traction in studying neurite outgrowth dynamics owing to their capabilities in real-time imaging and analysis. An analysis of 45 publications utilizing IncuCyte systems revealed trends in the experimental conditions, including the choice of well plate, cell density, neuron type, correlative experiments, microscopy techniques, and imaging duration. Most notably, 96-well plates were the most often used well plates, with 56% of the publications utilizing them in assays. However, despite the clarity of most of these publications when it came to reporting the parameters used in their studies, there were a few that did not report these experimental parameters, which we considered relevant for reproducibility. The transparency of these experimental designs is critical for the advancement of live-cell imaging instruments for neurite growth quantification assays. Reporting the specific parameters can help other labs establish, execute, or modify these types of assays. We hope that future labs will report the entirety of their experimental procedures and standardize the reporting of this type of experiment.

Furthermore, a previous investigation by our lab indicated that the green fluorescence of IncuCyte was harmful to primary rat cortical neurons and that DRAQ5 was cytotoxic when used with the red fluorescence channel of IncuCyte. This cytotoxicity may also be due to the higher sensitivity of primary neurons compared to that of other cell types. Based on this discovery, only the first image in Figure 5B is reliable for determining viability in the context of the experiment because any subsequent images will include viability loss due to the reagent. Another observed limitation is the measurement of neurite morphology. Degraded neurites (as determined by visual inspection) are not clearly distinguished from intact neurites, as the NeuroTrack mask often tends to count the area or branching points of evidently degraded neurites, making this analysis sometimes unreliable in neurite degeneration experiments. To overcome this limitation, it is beneficial to manually adjust the mask parameters so that the mask is representative of several images from your experiments or of the correlative experiments performed. We also recommend using the phase-contrast capability over fluorescence due to the cell viability issues we have encountered. This may be the reason why some of the publications reviewed resorted to the conventional MTT, Cell Titer-Glo, and Ethidium homodimer assays instead of using the fluorescence component of the IncuCyte. Alternatively, more nuanced image analysis techniques have been outlined by Bellon et al., including advanced image analysis algorithms that allow for precise quantification of neurite length and morphology [107]. Incorporating these approaches can complement live-cell imaging, especially in the development of training sets for artificial intelligence (AI)-assisted approaches.

The IncuCyte system, despite its ease of use, automated capabilities, and suitability for long-term studies, has certain limitations compared to other live-cell imaging systems. One significant limitation is the type of microscopy used. The IncuCyte employs phase-contrast and one-photon fluorescence microscopy. In contrast, instruments like Leica’s Mica microhub, Agilent’s Cytation 10, and Zeiss Cell Discoverer 7 use confocal microscopy in tandem with widefield fluorescent microscopy, which provides higher resolution images due to its focused imaging capabilities. This makes these instruments superior for detailed cellular analysis and imaging clarity.

One of the most promising future directions of these automated systems, particularly the IncuCyte, is the integration of artificial intelligence and machine learning (ML). AI and ML algorithms can analyze imaging data more efficiently and accurately than traditional methods by identifying subtle patterns and correlations that may be missed by human observers. Importantly, the reproducibility crisis in scientific research has been highlighted extensively over the past 20 years, and this issue is a chief concern for the use of AI in drug discovery. AI/ML models are only as good as their training sets. Erroneous training of AI/ML models on data that are not robust and/or reproducible could actually slow efforts to improve public health. For this reason, the use of automated imaging systems will be of crucial importance in the coming decade to acquire standardized datasets, including real-time images as raw data. From this, significantly more information can be extracted compared to the historic fashion of disclosing datasets, which are often processed and presented in tablature form. Developing standardized protocols and formats will facilitate data sharing and collaboration across research institutions, leading to more reproducible and comparable results. Furthermore, creating centralized databases of live-cell imaging data can provide valuable resources for the research community, driving innovation and discovery.

The application of live-cell imaging in personalized medicine represents the ever-evolving state-of-the-art, particularly with its ability to acquire data in systems with high relevance to human health. As with all preclinical paradigms, there is no ‘on-size fits all’ approach. However, there has been a long-standing reliance on immortalized cell lines (such as PC-12 and SHSY-5Y) in preclinical CNS drug discovery. Such systems may be useful in specific circumstances to study the modulation of specific targets/target pathways if appropriate controls, benchmarks, and orthogonal assays are employed. In contrast, more recently, the use of induced pluripotent stem cells (iPSCs), which can be reprogrammed to differentiate into neurons carrying patient-specific genetic information, has gained much traction and is anticipated to more accurately recapitulate human diseases and could potentially lead to personalized medicine. The integration of standardized datasets harnessing real-time analysis of drug-induced neuronal morphological changes could lead to the development of predictive models for neuronal behavior and drug response, significantly accelerating the drug discovery process. By using patient-derived neuronal cultures and live-cell imaging to screen for drug responses, researchers can develop tailored therapeutic strategies for individual patients. This personalized approach has the potential to improve treatment outcomes and reduce adverse effects in CNS disorders. The integration of AI and ML, advancements in imaging technologies, standardization efforts, and applications in personalized medicine represent key future directions that will shape the field of live imaging for CNS drug discovery. We hope these forward-looking insights provide a valuable perspective on the innovative potential and future impact of live-cell imaging systems in neuroscience research.

## 5. Conclusions

Live-cell imaging systems offer invaluable insights into the dynamics of neurite outgrowth, thereby contributing to the discovery of novel therapeutics for CNS disorders. By leveraging neuronal cell cultures and advanced imaging technologies, researchers can unravel the complexities of CNS pathology and identify potential drug candidates with high accuracy and precision. Standardized reporting of experimental conditions and rigorous optimization of imaging protocols are essential to ensure the reproducibility and reliability of CNS drug discovery research. Hence, collaborative efforts within the scientific community to share data, standardize methodologies, and validate findings, which will further accelerate progress in the field of CNS drug discovery, should be encouraged.

## 6. Experimental Methods

### 6.1. Reagents

IncuCyte^®^ NucLight Rapid Red Reagent (EssenBioScience Ann Arbor, MI, USA, Cat. 4717), and DraQ5 (Thermo Scientific, Waltham, MA, USA, Cat. 62251) were used, as directed by the manufacturers.

### 6.2. Neuron Cultures

Prepare *Sprague-Dawley* E18 rat cortical neurons, as described by the supplier (BrainBits, Springfield, IL, USA, Cat. SDED), and plated at 5000 cells per well per 100 mL of media in 60 inner wells of a 96-well poly-D-lysine coated plate (Corning Biocoat, Corning, NY, USA Cat. # 354413). To prevent evaporation, add 150 mL of Sterile DPBS (Gibco, Grand Island, NY, USA, Cat. # 14190-144) to the outer wells. Suspend the cells initially with BrainBits NbActiv media (Cat# NbActiv1 500) and count the cells using a hemocytometer and Gibco Neurobasal plus media (Cat. # A35829-01) supplemented with B27plus (Gibco Cat. # A35828-01), L-glutamine (Gibco Cat. #25030081) and pen/strep (Gibco Cat. #15140122) to bring the cells to an appropriate plating density. Incubate at 37 °C and 5% CO2. Do not move the plates for at least 4 h after plating to allow for an even spread of the cells as they attach to the plate. Feed the cells every 2-3 days (preferably 2) by first removing 50 mL of media and replacing it with fresh media. Start the experiments on day 9 of neuron culture. BrainBits animal protocol #32-08-013 was approved by the Southern Illinois University School of Medicine Laboratory Animal Care and Use Committee on 18 May 2011.

### 6.3. Live-Cell Imaging

Using the IncuCyte S3 Live Cell Analysis System (EssenBioScience), pre-scan the plate(s) immediately prior to experimentally manipulating the cultures. These results are used as a baseline for normalization of measurements to be performed after treatments. Scan settings are set for phase contrast at a magnification of 20× with the acquisition of 9 micrographs per well at 6 h intervals. Neurotracker analysis using Incucyte software version 2020B with a Filtering set at “best”, neurite sensitivity at 0.25, and neurite width set at 1 mM. Pre-scanning will also allow you to cull cultures that are uneven, contaminated, or have non-optimal neurites.

## Figures and Tables

**Table 1:** General features of live-cell imaging instruments.

Manufacturer	Instrument	Features
Leica Microscope Systems	Mica microhub [23]	4-color widefield fluorescence imaging Confocal microscopy Automated microscope Incubator for cell viability
Sartorius	IncuCyte [24]	5-color Fluorescence imaging channels. Phase-contrast microscopy. Automated microscope. Incubation up to 42 °C.
Agilent	Cytation 10 [25]	Widefield and Spinning disk confocal fluorescence microscopy. Brightfield and phase-contrast microscopy. Automated live-cell incubation.
	Lionheart FX [25]	Widefield fluorescence imaging. Brightfield and phase-contrast microscopy. Automated live-cell incubation.
	xCELLigence RTCA eSight [26]	Brightfield microscopy. Three fluorescence imaging channels.
	Biospa [27]	Fluorescence brightfield, color brightfield, and phase-contrast microscopy. Incubation up to 45 °C. Multi-component environmental control.
Molecular Devices	ImageXpress pico [28]	Imaging modes: phase contrast and brightfield, fluorescence, widefield, colorimetric, and confocal imaging.
Etaluma	Lumascope [29] (LS720)	3-fluorescence imaging channels. Brightfield and phase-contrast microscopy. External incubation is required.
Logos Biosystems	Celena S [30]	3-fluorescence imaging channels. Brightfield and phase-contrast microscopy. Onstage incubation System.
Axion Biosystems	Cytosmart Lux 3 [31]	2-fluorescence imaging channels. Brightfield microscopy. Incubation up to 40 °C.
Zeiss	Cell Discoverer 7 [32]	Brightfield, confocal, and widefield fluorescence microscopy. Temperature and atmospheric control features.
Keyence	BZ-X800 [33]	Fluorescent, brightfield, and phase-contrast microscopy. Built-in dark room. Time-lapse incubation.

**Table 2:** Summary of 45 publications that utilized IncuCyte systems in their neurite outgrowth assays.

Articles	Types of Plates	Density Plated	Type of Cells Used	Correlative Experiments Used	Disease State/Aim	Microscopy	Duration of Exposure	Other Imaging Instruments Used
Camarena, 2014 [53]	6-well	750,000 cells/well	Mouse Cortical neurons (Primary)	Immunocytochemistry	2q23.1 microdeletion syndrome	Phase-contrast	Q 3 h/48 h	LSM710 Zeiss confocal microscope
Cavaliere, 2017 [54]	96-well	10,000 cells/cm^2^	Rat Cortical neurons (Primary)	Immunocytochemistry	Parkinson’s Disease	Phase-contrast	Did not mention	IncuCyte
Efthymiou, 2014 [44]	96-well	10,000 cells/well	Stem cells (iC23-GFP NSCs)	Immunocytochemistry	Deriving neurons from pluripotent stem cell	Phase-contrast and Fluorescence	Q 2 h/14 days	Leica fluorescence microscope
Hong, 2018 [55]	96-well	5000 cells/well	induced Neurons from Stem cells (hIPSCs)	Immunocytochemistry	Alzheimer’s	Phase-contrast	Q 2 h/84 h (Baseline 2 h/6 h)	Zeiss LSM710 confocal microscope
Jin, 2018 [56]	96-well	not mentioned	hIPSCs	ELISA	Alzheimer’s	Phase-contrast	Q 2 h/3 days (Baseline 2 h/6 h)	Not applicable
Kobayashi, 2018 [57]	not mentioned	not mentioned	Retinal Ganglion cells from Embryonic Stem cells	Immunocytochemistry	Glaucoma/Blindness	Phase-contrast	Q 6 h/450 h	Confocal microscope (LSM700; Carl Zeiss
Korecka, 2019 [58]	96-well	15,000 cells/well	Stem cells (hIPSCs)		Parkinson’s Disease	Phase-contrast (implied)	24 h	IncuCyte
Laferrière, 2019 [59]	96-well	20,000 calls/well	Mouse Cortical neurons (Primary)	Immunocytochemistry	ALS/FTLD	Phase-contrast and Fluorescence	6 days	Life Technologies EVOS FL Auto imaging system
Li, 2018 [60]	96-well	50,000 cells/well	hIPSCs	Western Blot	Alzheimer’s (antibodies)	Phase-contrast	Q 2 h/3 days	Not Applicable
Li, 2017 [61]	48-well	200,000 cells/well	Rat Spinal Cord neurons (Primary)	Immunocytochemistry/Western Blot	Spinal Cord Injury	Phase-contrast and Fluorescence	Q 4 h/4 days	Olympus BX-60 epifluorescent microscope
Medina-Cano, 2018 [62]	24-well	not mentioned	Mouse Cerebellar granule cells (Primary)	Western Blot	N-glycosylation defect in brain development	Phase-contrast (implied)	Q 3 h/21 h	Not applicable
Mengel, 2019 [63]	96-well	5000 cells/well	hIPSCs	Western Blot	Alzheimer’s	Phase-contrast	Q 2 h/3 days)	Not applicable
Mohtaram, 2015 [64]	24-well	not mentioned	Cancer cells (PC-12)	Bioactivity assay	Delivery of GD neurotrophic factor for the treatment of CNS disorders during injury	Phase-contrast	Q 12 h/10 days	IncuCyte
Park, 2017 [65]	24-well	not mentioned	Glioblastoma Stem cells (Cancer Stem Cells)	Immunocytochemistry	ASCL1 suppresses tumorigenicity of glioblastoma	Phase-contrast	Q 4 h/10 days	Leica STP-6000 microscope and Leica SP8 Confocal microscope
Peng, 2017 [66]	8-well chamber slides	not mentioned	Human Sensory neuron	Immunocytochemistry	Protection of the PNS by a cytokine	Phase-contrast	Q 1 h/16 h 74 h after plating and treatment	Not mentioned
Robinson, 2017 [67]	not mentioned	not mentioned	Stem cells (hIPSCs)	Immunocytochemistry/Cell viability assay MTT	Deriving neurons from pluripotent stem cell	Phase-contrast	Not Mentioned	Leica DMI 30000B
Robinson, 2015 [68]	not mentioned	not mentioned	Stem cells (hIPSCs)	Immunocytochemistry	Parkinson’s Disease	Phase-contrast	Not clear	Leica DMI 30000B
Schwaid, 2018 [69]	96-well	2000 cells/well	Rat Dorsal Root Ganglion Neurons (Primary)	Mass Spectrometry (proteomics)	Comparison of rat and human proteome	Phase-contrast (implied)	7 days	Not applicable
Snyder, 2018 [70]	96-well	not mentioned	Stem cells (hIPSCs)	Immunocytochemistry	Neurotoxicity during drug development	Phase-contrast and Fluorescence	Q 12 h/3 days	LSM 710 confocal microscope (Zeiss)
Song, 2018 [71]	not mentioned	not mentioned	Mouse Ventral Mid-brain Neural Progenitor cells (Primary cell)	Immunocytochemistry	Parkinson’s Disease	Fluorescence	42 h	confocal microscope (Leica PCS SP5)
Soppa, 2014 [48]	24-well	90,000 cells/well	Cancer cells (SH-SY5Y)	Immunocytochemistry	Down Syndrome	Phase-contrast and Fluorescence	4 days	Axiovert 200 M inverted microscope (Zeiss)
Srikanth, 2018 [42]	96-well	15,000 cells/well	hIPSCs	Immunocytochemistry	Neuropsychiatric diseases	Phase-contrast	3 days	Zeiss LSM710
Subedi, 2016 [72]	6-well	10,000 cells/well	N2a (Mouse neuroblastoma cell line) Cancer cells	Cell viability assay MTT	Neuroprotective abilities of *Lindera neesiana*	Phase-contrast	24 h	Not applicable
Subedi, 2017 [73]	12-well	600,000 cells/well	N2a (Mouse neuroblastoma cell line) Cancer cells	Cell viability assay MTT/Western Blot	Neuroprotection due to Equol	Phase-contrast (implied)	Q 2 h/24 h	Not applicable
Tortoriello, 2014 [74]	not mentioned	25,000 cells/well	Mouse Primary cortical neurons	Western Blot	Substance abuse’s effect on children	Phase-contrast (implied)	Q 2 h/62 h	Not applicable
Venkatesan, 2016 [75]	24-well	50,000 cells/well	N2a (Mouse neuroblastoma cell line) Cancer cells	Cell viability and NGF assay	Lactucopicrin neuroprotective effect against scopolamine	Phase-contrast	24 h	Not applicable
Walsh, 2018 [76]	96-well	5000 cells/well	hIPSCs	Not applicable	Alzheimer’s	Phase-contrast (implied)	Q 2 h/4 days	Not applicable
Wang, 2018 [77]	48-well	200,000 cells/well	Rat Spinal Cord neurons (Primary)	Immunocytochemistry and ELISA	Spinal Cord Injury	Phase-contrast and Fluorescence	Q 4 h/5 days	Zeiss confocal microscopy
Woo, 2014 [78]	6-well	10,000 cells/well	N2a (Mouse neuroblastoma cell line) Cancer cells	Cell viability and NGF assay	Neuroprotective effect of *Dioscorea nipponica*	Phase-contrast	72 h	Not applicable
Yagi, 2015 [79]	96-well	6000 cells/well	Mouse Primary motor neurons	Cell viability assay MTS/Western Blot	Nerve regeneration due to Zonisamide	Phase-contrast	Q 8 h/3 days	Not applicable
Zhang, 2018 [80]	not mentioned	not mentioned	HT22 (mouse hippocampal cell line) immortalized cells	Western Blot	Differentiation of an immortalized cell line	Phase-contrast (implied)	24 h	Not applicable
Zhao, 2014 [81]	96-well	2500 cells/well	BE(2)-C Human Neuroblastoma cell line Cancer cells	Cell viability/Western blots	Neuroblastoma	Phase-contrast	Q 6 h/5 days	Not applicable
Zhao, 2016 [82]	96-well	not mentioned (but can be implied)	BE(2)-C Human Neuroblastoma cell line Cancer cells	Cell viability/Western blots	Neuroblastoma	Phase-contrast (implied)	4 days	Not applicable
Zhao, 2015 [83]	96-well	2500 cells/well	BE(2)-C Human Neuroblastoma cell line Cancer cells	Cell viability/Western blots	Neuroblastoma	Phase-contrast	Q 6 h/4 days	Not applicable
Zhao, 2018 [84]	96-well	2500 cells/well	BE(2)-C Human Neuroblastoma cell line Cancer cells	Cell viability/Western blots	Neuroblastoma	Phase-contrast	Q 12 h/4 days	Not applicable
Davies, 2022 [46]	96-well (day 6 neurons)	10,000 cells/well	iPSC-derived cortical neurons (stem)	Mass spec-based lipidomics, Western blot	AP-4 deficiency	Phase-contrast	Q 3 h/21 h	Not applicable
Tripathi, 2022 [85]	96-well	15,000 cells/well	Rat Cortical Neuron transfected with pLVX-IRES-mCherry plasmids (primary)	Immunocytochemistry	Synucleinopathies, e.g., Parkinson disease	Fluorescence	72 h after transfection	Imaging-based toxicity assay “mCherry coexpression”
Bartlett, 2022 [86]	24-well	50,000 cells/well	NSC-34 spinal cord × Neuroblastoma hybrid cell line	ER Stress Assay	ALS	Fluorescence	not mentioned	Leica DM IBRE
Deng, 2022 [87]	8-well	not mentioned	Neural stem cell	Immunocytochemistry	Autism	Phase-contrast	4 days	Leica SP5 confocal microscope
Baytas, 2021 [88]	96-well	25,000 cells/well	Mouse Hippocampal neurons (primary)	Immunocytochemistry	Postnatal growth defects	Phase-contrast	16 days	Olympus FV3000 confocal laser scanning microscope
You, 2022 [89]	96-well	not mentioned	SH-SY5Y P301L cells (cancer)	Western blotting	Alzheimer’s	Fluorescence	Q 15 min/1.5 h	Not applicable
Smith, 2023 [90]	96-well	5000 cells/well	NG108-15 (Neuroblastoma and glioma hybrid	Immunocytochemistry	Peripheral Neuropathy	Phase-contrast and Fluorescence	Q 4 h for 72 h	IncuCyte S3
Zosen, 2023 [43]	96-well	15,000 cells/well	Cancer cells (SH-SY5Y)	Western blotting/Immunocytochemistry	Depression	Phase-contrast	Q 4 h for 48 h	IncuCyte ZOOM
Sum, 2023 [91]	96-well	15,000 cells/well	Cancer cells (PC-12)	Cell viability assay MTT	neurotrophic activities of the drimane-type molecules	Phase-contrast	48 h	Not applicable
Sum, 2023 [92]	96-well	15,000 cells/well	Cancer cells (PC-12)	Cytotoxicity assay	neurotrophic activities of the Hericioic acids	Phase-contrast	48 h	Not applicable

## Data Availability

The original contributions presented in the study are included in the article, and further inquiries can be directed to the corresponding author.
